# Risks to mental health of higher degree by research (HDR) students during a global pandemic

**DOI:** 10.1371/journal.pone.0279698

**Published:** 2022-12-27

**Authors:** Charlotte Brownlow, Douglas Eacersall, Charles W. Nelson, Renée L. Parsons-Smith, Peter C. Terry

**Affiliations:** 1 Graduate Research School, University of Southern Queensland, Toowoomba, Australia; 2 Centre for Health Research, University of Southern Queensland, Toowoomba, Australia; 3 Library Services, University of Southern Queensland, Toowoomba, Australia; 4 School of Psychology and Wellbeing, University of Southern Queensland, Toowoomba, Australia; 5 School of Health and Behavioural Sciences, University of the Sunshine Coast, Sippy Downs, Queensland, Australia; University of Johannesburg, SOUTH AFRICA

## Abstract

The COVID-19 pandemic has affected university students globally. Our study investigated mental health indicators among higher degree by research (HDR) students at a regional university in Queensland, Australia. A total of 231 HDR students (female = 137, male = 94) completed the Brunel Mood Scale to assess the constructs of Tension, Depression, Anger, Vigor, Fatigue, and Confusion. A subset of 11 students participated in three focus groups to explore their experiences. Results showed that reported mood among HDR students was generally more negative than population norms, although more positive than moods reported previously during the pandemic. A total of 52 participants (22.5%) reported mood profiles that indicated elevated risk of mental ill-health. Mood profiles varied significantly by gender, age, study mode (full-time/part-time), location (on-campus/online), and citizenship (domestic/international). Quantitative data were supported by focus group findings, which identified mental health and wellbeing as key themes of concern to HDR students. Our findings indicate that support mechanisms to safeguard the mental health and wellbeing of HDR students should be a priority for universities.

## Introduction

University students globally have been adversely affected by the COVID-19 pandemic [1]. Mental health indicators, in particular, have demonstrated the emotional toll on students. For example, Ma and colleagues reported a prevalence rate for acute stress, depression, and anxiety symptoms of 34.9%, 21.1%, and 11.0%, respectively among a sample of 746,217 college students in China during the COVID-19 outbreak [2]. Similarly, a survey of students at a large American public university [3] showed that 71% reported increased stress and anxiety due to the COVID-19 outbreak. Specific stressors included fear and worry about their own health and of their loved ones (91%), difficulty in concentrating (89%), disruptions to sleeping patterns (86%), decreased social interactions due to physical distancing (86%), and increased concerns about academic performance (82%) [3].

For higher degree by research (HDR) students, the demands of undertaking a research degree can be stressful and challenging even in the best of times [4–8]. Reported stressors include financial pressures, a lack of institutional support, inadequate training initiatives, administrative processes [4], difficulties establishing a researcher identity [5, 6], the pressures of meeting milestones, such as confirmation of candidature [7], as well as struggling with feelings of social isolation, lack of motivation, and challenges with supervisors and the academic environment [8]. The challenges of research study often affect the mental health of HDR students [4], with more than 40% reporting “depression … or high levels of stress” [9] during their studies.

A comprehensive review by Sverdlik et al. [10] of 163 empirical studies that had investigated factors influencing the mental health of doctoral students highlighted the complexity of the doctoral experience. The authors concluded that four factors external to the student (i.e., supervision, personal and social lives, departmental support and socialization, financial opportunities) and three internal factors (i.e., motivation, writing competencies, academic identity) interact to shape the overall experience of doctoral study and the attendant risk to mental health.

The COVID-19 pandemic has resulted in additional impacts for HDR student wellbeing. Although some research has recorded positive effects for doctoral researchers, including increased work-life balance and better productivity due to work from home arrangements [11], the majority of findings have shown negative impacts for research students as a result of COVID-19. This has included increased anxiety and stress [12, 13], poorer general health and academic functioning [14], and disruptions to overall wellness [15], mental health [16], and mood [14].

Collectively, these impacts represent a significant deleterious effect on the mental health and associated wellbeing of HDR students. For example, Anwer et al. [12], in a study of 108 full-time postgraduate students in Hong Kong, found most students reported high levels of poor sleep (83%), mild to severe levels of anxiety (76%), and moderate levels of stress (89%) during the pandemic. Similarly, Paucsik et al. [13] reported significant increases in stress, anxiety, and depression, and decreases in engagement and wellbeing of doctoral students in France, during a one-year period of the pandemic. Pyhalto et al. [16], in a mixed methods study of 768 Finnish PhD students, found that COVID19 had negatively impacted student progress and mental health, with the highest risk for full-time students, those studying in the natural sciences, and those in the middle stages of their degree.

Assessment of mood is commonly used to screen individuals for risk of mental health issues. The mood measure used in the present study, the Brunel Mood Scale [17, 18] includes subscales to assess Tension, Depression, Anger Vigor, Fatigue, and Confusion. According to the Mental Health Model [19] a mood profile characterized by a high score for Vigor and low scores for Tension, Depression, Anger, Fatigue, and Confusion is indicative of positive mental health, whereas a profile characterized by a low score for Vigor and high scores for Tension, Depression, Anger, Fatigue, and Confusion indicates risk of mental ill-health. The former profile has been termed the iceberg due to its shape when plotted graphically [19] and the latter profile is known as the inverse iceberg [20].

More recently, four other mood profiles have been identified in the literature [21]. The most negative of these, referred to as the inverse Everest profile is characterized by a low score for Vigor, high scores for Tension and Fatigue, and very high scores for Depression, Anger, and Confusion. The high to very high scores on negative mood dimensions indicate elevated risk of mental ill-health and are associated with a range of psychological disorders [22–24]. The shark fin profile, which is characterized by below average scores for Tension, Depression, Anger, Vigor, and Confusion, combined with very high Fatigue scores, has been linked to athletic injury [25] and poor adherence to safety procedures in high-risk vocations [26]. The surface profile, which is characterized by average scores on all mood dimensions, can be considered to represent a typical mood, and the submerged profile, which is characterized by below average scores on all mood subscales, may be beneficial in activities that place a premium on remaining calm and unemotional [27].

Mood profiling is used globally to assess for risk of mental health issues among a wide variety of populations. Examples include screening for risk of post-traumatic stress disorder among military personnel in South Africa [24]; evaluating population-level mental health and monitoring the psychological wellbeing of cardiac rehabilitation patients in Brazil [28, 29]; managing performance anxiety and preventing injuries among ballet dancers in Japan [30] and assessing youth for elevated suicide risk in the USA [31].

The aims of the present study were (1) using quantitative methods, to compare the moods of HDR students with pre-COVID population norms [n] and the moods of a population sample assessed during the pandemic [n]; (2) to establish whether six distinct mood profile clusters identified in the general population [n] were also evident among HDR students, their relative prevalence, and the associated risk of mental ill-health; and (3) using qualitative methods, to explore the experiences of HDR students during the period of the pandemic.

## Methods

The present study used a sequential mixed-methods approach, with an initial online survey followed by a series of focus groups among HDR students to better understand their experiences. The survey therefore informed the development of the discussion points and participant composition for the focus groups. The reporting structure for this mixed-methods study is informed by the recommendations of Tariq and Woodman [32], whereby the two parts of our study are reported separately in our methods and results sections and then integrated in our discussion section.

### Part 1: Online survey

#### Participants

A heterogeneous sample of 231 HDR students, 137 of whom identified as female and 94 as male, completed an online survey. In the context of Australian universities, HDR students are those students undertaking research degrees at masters (e.g., Master of Research) or doctoral level (e.g., Doctor of Philosophy). Participants were born either in the 1940s (*n* = 10), 1950s (*n* = 23), 1960s (*n* = 59), 1970s (*n* = 61), 1980s (*n* = 58), or 1990s (*n* = 20). A total of 184 were domestic students and 47 were international students; English was the first language of 160 participants and 71 had a different first language; 102 were first in family to attend university and 129 were not; 109 studied on-campus and 122 studied online; 96 were full-time students, 89 were part-time students, and 46 has switched between full-time and part-time mode during their studies.

#### Measures

*Brunel Mood Scale (BRUMS)*. The BRUMS [17, 18] includes 24 mood descriptors to assess six dimensions of mood (i.e., Tension–items nervous, anxious, worried, panicky; Depression–items unhappy, miserable, depressed, downhearted; Anger–items bitter, angry, annoyed, energetic; Vigor–items energetic, active, lively, alert; Fatigue–items exhausted, tired, worn out, sleepy; and Confusion–items mixed up, muddled, uncertain, confused). Respondents indicate how they are feeling “right now” on a 5-point Likert-type scale anchored by 0 = not at all and 4 = extremely. Scores for each mood subscale range from 0–16.

All subscales have shown satisfactory internal consistency, with Cronbach alpha coefficients ranging from .74 to .90 [17, 18]. In the original validation studies, the BRUMS demonstrated robust psychometric properties using multi-sample confirmatory factor analysis that supported the configural, metric, scalar, and residual invariance of the measurement model across samples of adult students, adult athletes, young athletes, and schoolchildren [17, 18]. In the present study, Cronbach alpha coefficients for the six mood subscales ranged from .83 to .93. Recent tables of normative data for the BRUMS based on the responses of 15,692 participants [33] informed the interpretation of our results.

#### Procedure

Following approval by the Human Research Ethics Committee of the host university (approval # H21REA024), email invitations to participate were sent by the Graduate Research School to 1,082 enrolled or recently graduated HDR students (21.3% response rate), with three follow-up email reminders. The survey was also advertised on the internal university HDR online forums and posters containing a QR link to the survey were displayed on campus. Participants were treated in accordance with the Australian Code for the Responsible Conduct of Research [34]. All participants were provided with an information sheet stating the purpose of the research at the start of the survey, with a check box indicating provision of informed consent to begin the survey. Final consent to participate was reflected in the submission of the survey by participants.

#### Data analysis

Quantitative data were compiled for analysis using SPSS for Windows, Version 28 [35]. Descriptive statistics for the BRUMS were calculated and group comparisons by demographic variables of interest (gender, age group, mode of study, etc.) were conducted using a series of one-way MANOVAs and *post hoc* pairwise tests. The alpha level for multivariate tests was set at *p* < .05 and, following a Bonferroni adjustment, at *p* < .008 for univariate tests involving the BRUMS subscale scores.

A seeded k-means cluster analysis was used to determine whether the six mood profiles previously identified in the literature, referred to as the iceberg, inverse Everest, inverse iceberg, shark fin, submerged, and surface profiles [21], were evident in the current sample. Chi-squared tests were used to determine whether the prevalence of specific mood profiles varied by demographic variable.

### Part 2: Focus groups

#### Participants

Eleven participants contributed to three online focus groups. Focus group 1 comprised four international HDR students and lasted 71 minutes. Focus group 2 comprised four HDR students who were studying part-time and lasted 62 minutes. Focus group 3 comprised three HDR students who were studying full-time and lasted 61 minutes. The focus groups included seven participants who identified as female and four who identified as male.

#### Procedure and protocol

The three focus groups were conducted to enhance our understanding of the experiences of HDR students. Following approval by the Human Research Ethics Committee (approval # H21REA024), advertisements for participants were distributed to HDR students via email and internal university online forums. On receipt of written informed consent, focus groups were conducted in accordance with a standard protocol [36] with discussion focused around the four topics of supports and challenges for HDR students, research culture, supervisory practices, and suggestions for change. A decision was taken to conduct homogenous focus groups with small numbers of participants in order to allow for shared experiences to be fully considered. The number of focus groups are in line with evidence for good practice identified by Guest et al who propose from their review that three focus groups were adequate to identify 80% of all themes prevalent within a dataset [37].

#### Data analysis

Thematic analysis supported by NVivo [38] was conducted on verbatim transcripts of focus group discussions following the six-step protocol of Braun and Clarke [39]. Following verbatim transcription, initial codes were identified which were then collated into thematic groups. The candidate themes were then reviewed by the author team and carefully checked against the raw data in order to ensure that they could be evidenced within the dataset. Following discussions within the research team, the themes were reviewed and defined. Extracts from the focus groups are included in order to support claims by empirical examples as evidence. Thematic analysis was conducted both within each distinct focus group and across all three focus groups to enable an understanding of experiences that were distinct to particular groups and those that were shared across groups.

## Results

### Part 1: Online survey

**Fig 1:**
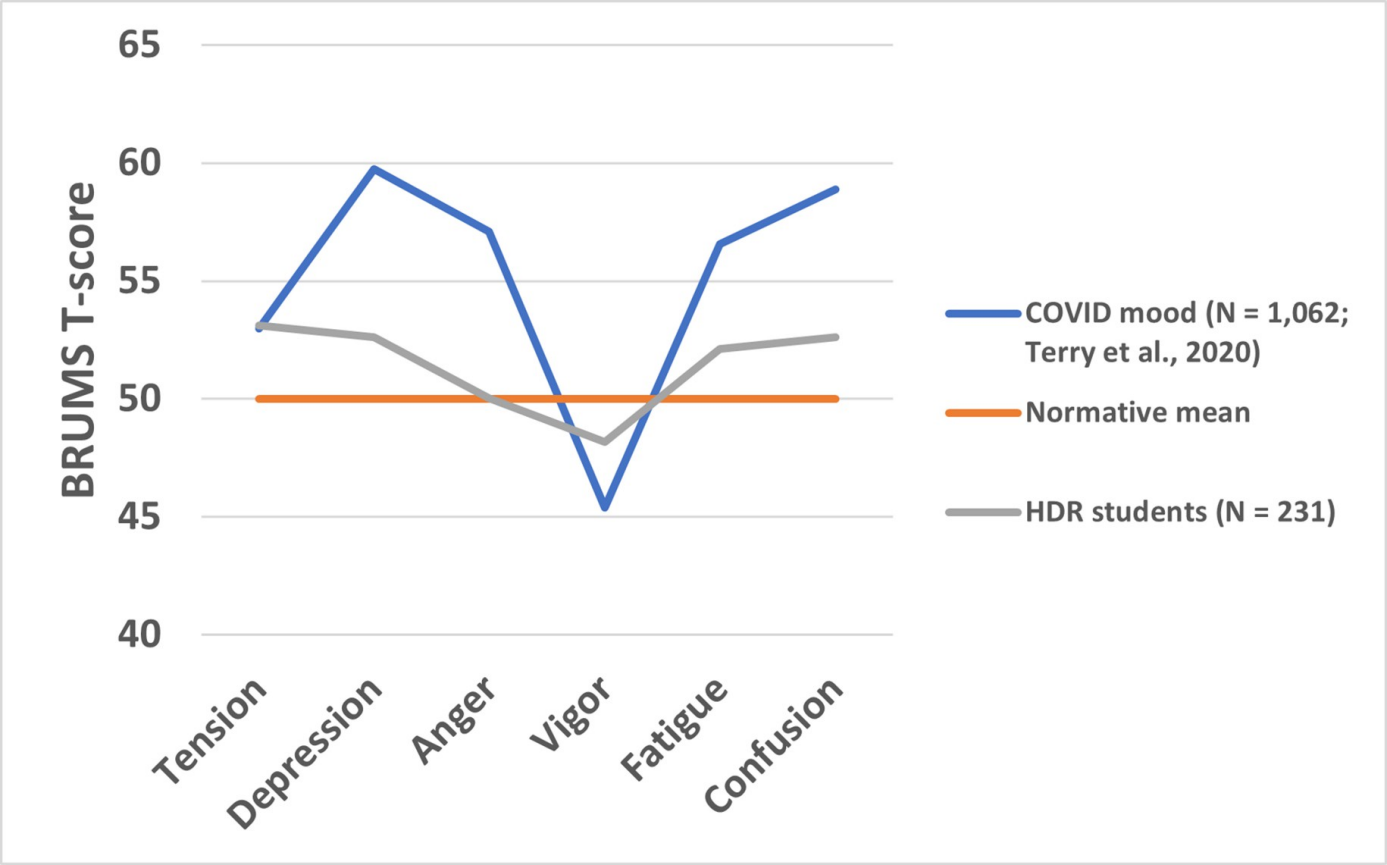
Mood scores of HDR students compared to BRUMS norms and COVID mood.

Mood scores are shown in Table 1. Standardized mean scores for BRUMS subscales for the whole sample varied from population norms (*M* = 50, *SD* = 10), ranging from 48.16 for Vigor to 53.12 for Tension. This pattern of mood scores is referred to as an inverse iceberg profile and is seen as indicating elevated risk of mental health issues [33, 40]. Fig 1 shows the mean scores for HDR students plotted against BRUMS norms. To provide an additional point of comparison, mean BRUMS scores gathered during COVID lockdown [41] are also plotted.

**Table 1:** BRUMS subscale scores for participants (N = 231) grouped by demographic variables.

Source	*n*	Tension	Depression	Anger	Vigor	Fatigue	Confusion	*F*	*η2*
Overall	231	53.12 (10.96)	52.62 (12.06)	50.04 (9.52)	48.16 (9.33)	52.12 (11.56)	52.60 (11.12)		
Male	94	51.02 (9.82)	51.43 (10.98)	49.54 (8.83)	49.91 (9.49)	**48.57 (10.19)**	51.45 (10.84)	2.83**	.082
Female	137	54.55 (11.49)	53.45 (12.73)	50.39 (9.99)	46.95 (9.06)	**54.55 (11.85)**	53.39 (11.29)		
Older^a^	92	**50.20 (9.64)**	50.32 (10.46)	48.39 (8.97)	48.95 (9.42)	**48.90 (10.77)**	50.23 (10.42)	2.17*	064
Younger	139	**55.05 (11.38)**	54.15 (12.83)	51.14 (9.75)	47.63 (9.27)	**54.24 (11.61)**	54.17 (11.33)		
Domestic	184	**52.02 (10.37)**	**51.57 (10.94)**	**49.03 (8.65)**	47.18 (9.17)	52.42 (11.46)	**51.46 (10.47)**	5.41^†^	.145
International	47	**57.40 (12.19)**	**56.74 (15.15)**	**54.00 (11.36)**	51.96 (9.05)	50.94 (12.00)	**57.06 (12.53)**		
Full-time^b^	96	**55.09 (11.90)**	54.61 (14.07)	51.92 (10.84)	49.26 (8.72)	51.09 (11.65)	**54.98 (12.06)**	4.02^†^	.119
Part-time	89	**50.71 (9.53)**	50.58 (9.29)	48.58 (7.64)	47.25 (9.32)	51.92 (11.30)	**49.39 (8.99)**		
On-campus	109	55.06 (11.97)	**55.05 (13.79)**	**52.04 (11.15)**	48.73 (9.66)	52.92 (11.46)	**55.43 (12.54)**	2.41*	.070
Online	122	51.38 (9.69)	**50.46 (9.84)**	**48.26 (7.39)**	47.64 (9.03)	51.40 (11.65)	**50.07 (9.23)**		

Note. Only comparisons yielding significant between-group differences are shown. BRUMS = Brunel Mood Scale, *η2* = partial eta-squared (% of variance explained by group membership). ^a^Older participants were born before 1970, younger participants were born from 1970 onwards. ^b^Participants who studied a combination of full-time and part-time were excluded from this analysis.

*p < .05

**p < .01

^†^*p* < .001. Pairs of scores in bold were significantly different at *p* < .008 (i.e., Bonferroni-adjusted)

Comparison of mood scores grouped by demographic characteristics showed significant differences between groups (Table 1). Females reported higher Fatigue scores than males. Younger students reported higher Fatigue and Tension scores than older students. International students reported higher Tension, Depression, Anger, and Confusion scores than domestic students. Full-time students reported higher Tension and Confusion scores than part-time students. Between-group differences explained between 6.4% (older vs. younger) and 14.5% (domestic vs. international) of the variance in mood scores (Table 1).

### Cluster analysis

**Fig 2:**
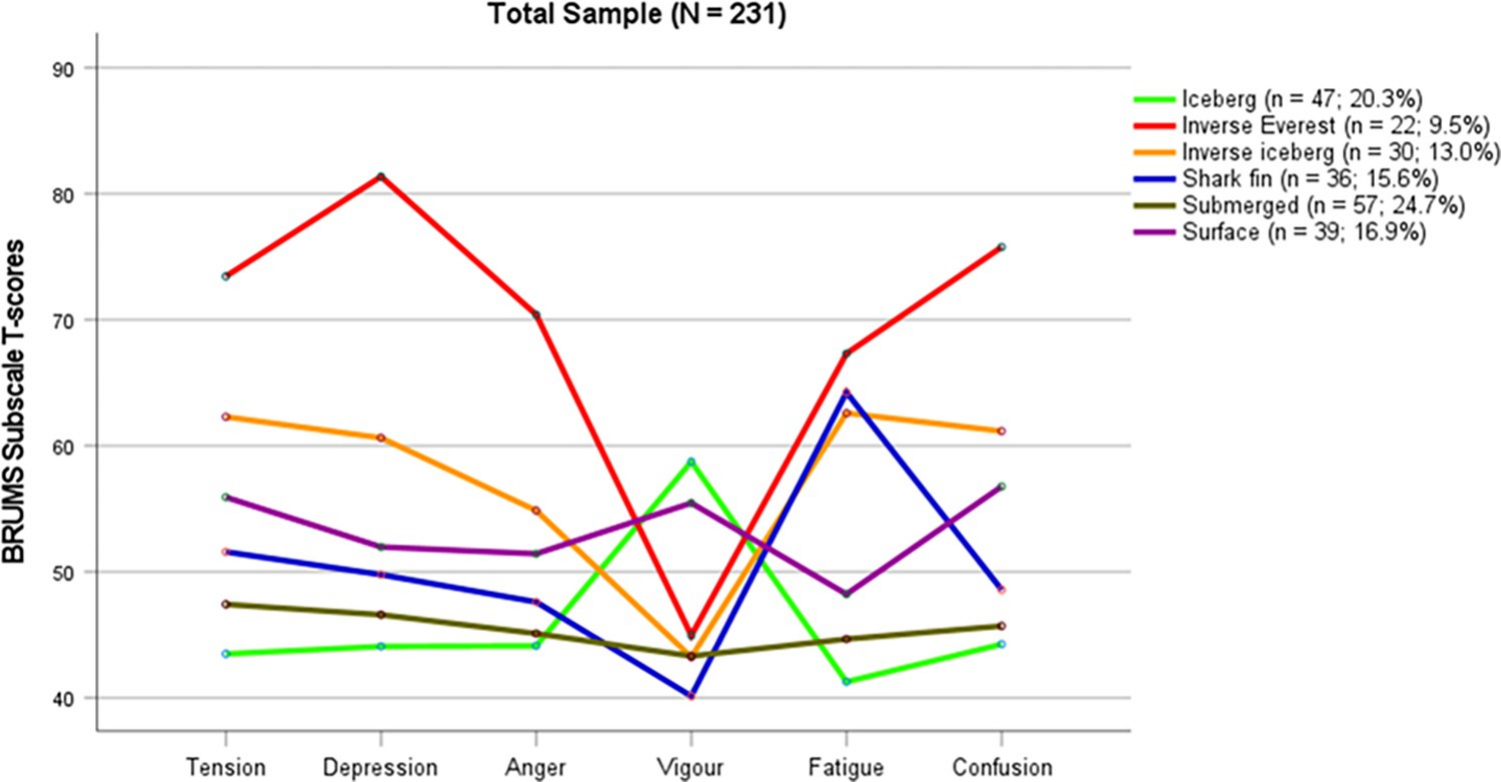
Graphical representation of the six-cluster solution.

Results of a seeded k-means cluster analysis showed that all six mood profiles described in the literature (i.e., iceberg, inverse Everest, inverse iceberg, shark fin, submerged, and surface profiles) were clearly distinguishable among our sample of HDR students. Fig 2 shows a graphical representation of the six profile clusters and their prevalence rates.

#### Cluster prevalence

Significant variations in cluster prevalence were found for several demographic variables (Table 2). For gender, females were overrepresented, and males underrepresented for the inverse iceberg and shark fin profiles, whereas males were overrepresented and females underrepresented for the submerged profile. Older participants were overrepresented and younger participants underrepresented for the iceberg profile. For study mode, full-time students were overrepresented and part-time students underrepresented for the inverse Everest profile and the surface profile, whereas part-time students were overrepresented and full-time students underrepresented for the shark fin profile.

**Table 2:** Distribution of clusters by demographic variables (N = 231).

Source	Cluster											
	1	%	2	%	3	%	4	%	5	%	6	%
Gender χ^2^(5, 231) = 14.89^§^												
Male (*n* = 94)	23	24.5	8	8.5	7*–	7.4	8*–	8.5	**31** * ^**+**^	33.0	17	18.1
Female (*n* = 137)	24	27.8	14	13.0	**23** * ^**+**^	17.8	**28** * ^**+**^	21.4	26*–	19.0	22	23.1
Age group χ^2^(5, 231) = 13.83*												
Older (*n* = 92)	**26** * ^**+**^	28.3	5	5.4	8	8.7	14	15.2	28	30.4	11	12.0
Younger (*n* = 139)	21*–	15.1	17	12.2	22	15.8	22	15.8	29	20.9	28	20.1
Citizenship χ^2^(5, 231) = 29.04^†^												
Domestic (*n* = 184)	38	20.7	**11**^†^–	6.0	26	14.1	**34** * ^**+**^	18.5	**51** * ^**+**^	27.7	**24**^**§**^–	13.0
International (*n* = 47)	9	19.1	**11** ^†^ ^**+**^	23.4	4	8.5	2*–	4.3	6*–	12.8	**15** ^**§**^ ^**+**^	31.9
Study mode χ^2^(5, 231) = 13.67*												
Full-time (*n* = 96)	18	18.8	**15** ^**§**^ ^**+**^	15.6	9	9.4	8*–	8.3	23	24.0	**23** * ^**+**^	24.0
Part-time (*n* = 89)	19	21.3	**3**^**§**^–	3.4	11	12.4	**19** * ^**+**^	21.3	26	29.2	11*–	12.4
Location χ^2^(5, 231) = 16.89^§^												
On-campus (*n* = 109)	21	19.3	**16** * ^**+**^	14.7	17	15.6	10*–	9.2	24	22.0	21	19.3
Online (*n* = 122)	26	21.3	6*–	4.9	13	10.7	**26** * ^**+**^	21.3	33	27.0	18	14.8

***Note*.** 1 = Iceberg, 2 = Inverse Everest, 3 = Inverse iceberg, 4 = Shark fin, 5 = Submerged, 6 = Surface; Bold font indicates significant

differences in cluster prevalence between groups; + = over-represented,— = under-represented

^†^*p* < .001

^§^*p* < .01

*p < .05.

For learning mode, on-campus students were overrepresented and online students underrepresented for the inverse Everest profile, whereas online students were overrepresented and on-campus students underrepresented for the shark fin profile. For citizenship, international students were overrepresented and domestic students underrepresented for the inverse Everest profile and surface profiles, whereas domestic students were overrepresented and international students underrepresented for the shark fin and submerged profiles.

### Part 2: Focus groups

Three key themes relating to mental health and wellbeing were identified from the focus group discussions. These were (1) feeling competent, (2) balancing life demands, and (3) isolation and peer support. Selected quotes from focus group (FG) member are included below to illustrate the three themes.

#### Theme 1: Feeling competent

Reflections on self-competence was a theme that ran through all three focus groups but was particularly evident in the discussions of the full-time students. A fear of writing was a commonly expressed concern:

But it is a fear of writing. You read someone’s work and you go, oh my God, that is brilliant, I can’t come up to that from scratch. Being able to sit down and write without feeling that stress, that tension, that burden and learning how to do it I think would be amazing. (FG3; Full-time student)

Frequently the fear was due to questioning their own expertise and not having both the confidence and ability to clearly articulate research ideas. For example:

There’s another thing that goes with that is I don’t consider myself an expert in this area yet, right? I don’t feel confident to be able to get up because I’m still discovering stuff…It’s not until ideas are properly formed and I can articulate them clearly in possibly a new kind of language that I’m ready for publication and conferencing. (FG2; Part-time student)

Feelings of self-competence were therefore central in students progressing their ideas through to submissions to journals and conferences within their field.

Issues of feeling competent were magnified in supervisory meetings, where students reported anxiety in preparing for supervision:

This year, particularly, I’ve had such high anxiety prior to meetings. It’s sort of like, oh my God, what am I going to do? I feel like I haven’t done enough. Anyway, after them is always a relief and I say to my kids, yeah, fluffed it through another one. (FG3; Full-time student)

In the example above the imbalance of power between the supervisor and the student was evident, with the student often reluctant to discuss their feelings of research incompetence with their supervisory team, preferring instead to create an impression for the supervisor that things were progressing well. Interestingly, there was an acknowledgement that such feelings should and could be normalized through interrogating expectations of selves, but this was not always drawn on by students. For example:

I think, too, is that sense of normalising. Because I think we just think everyone who’s in this position is going to be just awesome at writing and you’re just this outcast because you find it quite challenging. Just to normalise it in those sessions, too, would be probably a good thing. (FG3; Full-time student)

Feelings of competence were identified as an important aspect of managing HDR studies and contributions to mental health and wellbeing. Additional influences were cited in terms of managing extraneous demands that were unrelated to studies but can be considered to have an impact on the mental health and wellbeing of students.

#### Theme 2: Balancing life demands

HDR students contributing to this study were clear in pointing out the additional roles and tasks that they must juggle in addition to their research. This was particularly amplified for part-time students, who were frequently balancing employment and a family with their studies. When this was reflected on by full-time students, these tensions in roles and available resources to fulfil these was sometimes amplified. For example:

I’m a single mother, I have to work as well as do my PhD. Sometimes my supervisor would like me to come to campus, so for me that school drop off, drive an hour and a half, have a meeting, drive an hour and a half back, school pick up. It’s just too much for me, so working remotely is a good thing. (FG3; Full-time student)

Part-time students also reported similar tensions concerning available resources, both personal and financial, to allocate to their HDR studies:

It’s just you’ve got to allocate your resources and when you’re working and doing PhD and we’ve all got other stuff we’re trying do as well [42:30], it’s just not tenable. (FG2; Part-time student)

However, for part-time students the frequent allocations of personal resources to crucial things other than HDR studies were reported to have an impact on the fluency of the research process:

…the disjointed nature of part-time study is, you know, lack of efficiencies and you don’t get the synergies and you don’t get focus and planning and writing blocks and it just—you come back going what did I do there and then you’ve got to start again. It’s just very, very inefficient. (FG2; Part-time student)

For students studying during the global pandemic, additional strains were felt to compound the research that were beyond the student’s control but were felt to have significant impact on progress and planning. For example:

I’m a single mum with two kids; just trying to fit everything in [19:00] is just really difficult…. I also had a lot of difficulty during COVID with data collection as I was looking at interviewing teachers and for 18 months, we weren’t allowed to talk to any teachers. So COVID probably put a stop to that which made it quite difficult. (FG2; Part-time student)

The unpredictability of external demands on students were therefore considered to have an impact on the experiences of the students, and the absence of feelings of support was raised as a key influence on the mental health and wellbeing of HDR students.

#### Theme 3: Isolation and peer support

Feelings of isolation and the need for peer support was discussed by all the focus groups but was particularly amplified for international students. For example:

Greatest challenge for me as a HDR student and as an international student—being alone here, it’s actually…being away from home. (FG1; International student)

However, international students were not the only ones who reported the impacts of being isolated, with COVID cited by many as a key influence on their experiences of social connectedness with others, as one student described it the “Lack of contact with actual people.” (FG3; Full-time student). For example:

I’m full time, supposed to be on campus but due to COVID and so forth, I’m working from home. What I’ve found is, because I’m not actually interacting with people on a daily basis, I’ve found that quite isolating. (FG3; Full-time student)

That isolation from peers. You’ve got the person sitting next to you and it’s like, hey, I’m thinking about blah, blah, blah, what do you think of that? They’ll go, yes, no, or I do this and then I can move forward. I guess I was only on campus for probably only a few weeks before we had our lockdown, so it was really great in that short period of time. There was only a handful of us in the HDR room but we all got on…if any of us had a question we’d sit down and we’d talk about it and then go back to our work, which was great. (FG3; Full-time student)

It was therefore apparent that HDR students’ feelings of isolation were compounded by both individual circumstances and issues beyond their control. Students did however report actions to address feelings of isolation and an attempt to promote feelings of social support, but these were sometimes met with frustrations and barriers. For example:

I think for me, I’ve probably felt isolated a lot… I should try and reach out and a couple of attempts that I’ve done to do that didn’t really go anywhere. (FG2; Part-time student)

However, in the absence of face-to-face or more individual support, other students were harnessing the possibilities for virtual connection through groups facilitated by the university. For example:

Also, I find the HDR Facebook group—there is a [university] HDR Facebook group, which is just nice to, I guess, kick me—give me a kick up the butt too just whenever scrolling Facebook going oh, that’s right, that’s there. That’s a really supportive group as well that often has some really good info. So that’s kind of my team, I guess. (FG2; Part-time student)

It was clear from the focus groups that students shared common discussions of the challenges posed to mental health and wellbeing through issues of personal belief in self-competence, the challenges of balancing life demands, which were frequently beyond their control, and the importance of connection with other students.

## Discussion

As a group, our sample of HDR students reported moods that, in terms of mental health, indicated they were more at risk than the general population under normal circumstances but less at risk than during COVID-19 induced lockdown [41, 42]. Scores for negative aspects of mood tend to be positively skewed, with a large proportion of the population scoring at or close to the minimum with a much smaller number scoring towards the upper end of the scale; and this was indeed the case with our sample. As a result, most participants reported mood scores below the population mean for Tension, Depression, Anger, Fatigue, and Confusion, suggesting a reduced risk of mental health issues, with only a minority of participants in the elevated risk category.

However, it is evident that 52 participants (22.5%) reported an inverse Everest or inverse iceberg profile, meaning that between 1 in 4 and 1 in 5 of the HDR students in our sample were at risk of experiencing mental health issues. This prevalence of the most negative mood profiles is higher than for the general population, where typically 15.7% of people (about 1 in 6) report either an inverse Everest or inverse iceberg profile [33]. On a more positive note, our sample compared favourably with mood profiles gathered during pandemic-induced lockdowns, which indicated that about 1 in 3 people reported increased risk of mental ill-health [41].

Between-group comparisons showed that the characteristics of HDR students most associated with risk of mental ill-health and reduced psychological wellbeing are female, younger, international, full-time, and on-campus. Aristovnik and Keržič [1], in a global survey of 30,383 undergraduate students from 62 countries, similarly found that females studying full-time were generally affected more by the pandemic than other groups in terms of their emotional life and personal circumstances.

Moods have been shown to vary according to gender, with men tending to report higher Vigor and lower Tension, Depression, Anger, Fatigue, and Confusion scores than women [40]. Moreover, the prevalence of mood disorders in Australia is substantially higher in women than in men [43]. Gender comparisons in our sample showed that female HDR students reported significantly higher Fatigue scores (Table 1) and were more than twice as likely to report an inverse iceberg or a shark fin profile than male HDR students (Table 2). Biological explanations for mood differences between men and women typically emphasize hormonal and reproductive functions [44, 45], although there are many socio-psychological reasons why women might report more negative moods than men, especially related to the disadvantage women face in several aspects of life, such as education, family responsibilities, and careers [46, 47].

Age-related differences in mood profiles were also evident, with younger HDR students reporting significantly higher Tension and Fatigue scores than older HDR students (Table 1). Moreover, they were twice as likely to report the most negative profiles (i.e., inverse Everest and inverse iceberg) and half as likely to report the most positive iceberg profile, compared to older HDR students (Table 2). Similar age-related differences in mood have been found among the general population, both prior to [33] and during the pandemic [41], which have been explained in terms of adaptive coping strategies being more common and better developed among older adults compared to their younger counterparts [48, 49].

In terms of citizenship, international HDR students were about four times more likely to report an inverse Everest profile compared to domestic HDR students. Although international students face similar challenges to domestic students in terms of completing an HDR degree, they experience additional stressors as temporary migrants and are at increased risk of experiencing poor mental health, with isolation from families and culture, language barriers, financial stress, and academic pressures among the key drivers [50]. Further, international students have been found to be less likely to seek help for mental ill-health than domestic students [51].

Study mode was also shown to be related to mood profiles, with full-time HDR students reporting significantly higher Tension and Confusion scores (Table 1) and being five times more likely to report an inverse Everest profile compared to part-time HDR students (Table 2). Qualitative data illustrated that both full-time and part-time students feel the stress of balancing the demands of life and study, although logically the stress may be more acute for full-time students who have less non-study time available to devote to other responsibilities. Part-time students may find it easier to maintain a sense of normality outside of HDR study and have access to a more extensive social support network than full-time students operating in an intense study environment, which may provide a buffer against the stressors involved [52].

Similarly, study location was linked to mood responses, with on-campus HDR students reporting significantly higher scores for Depression, Anger, and Confusion (Table 1), and being three times more likely to report an inverse Everest profile, compared to HDR students studying online (Table 2). Although some evidence implicates online study as contributing to increased social isolation and threats to mental health [1, 2, 53–55] in situations where HDR students out of necessity must balance the demands of family responsibilities with the opportunity for higher degree study, the greater flexibility afforded by online study may alleviate rather than exacerbate the stressors involved, as suggested by the present findings. It should be noted that the university in which the present study was conducted has a long history of providing successful online education, and hence the findings related to study location may not generalize more broadly.

The qualitative data helped to unpack the survey results by providing insights into some of the specific stressors experienced by HDR students that might be responsible for the increased risk of mental ill-health indicated by mood profiles. Previous evidence has shown that doctoral students struggle with a wide range of stressors that include social isolation, time pressure, lack of motivation, uncertainty of doctoral processes, financial concerns, doubts about academic competence, work-life balance, and challenges with supervisors [8]. The results of our focus groups endorsed all of these stressors, whilst highlighting the three themes of feeling competent, balancing life demands, and isolation and peer support. It was clear from all three focus groups that individual stressors associated with a questioning of own competence was challenging for students. The typical support mechanisms were frequently not in place to mediate this, particularly for international students. Furthermore, the challenges of balancing other life tensions were clearly articulated by the participants, indicating that the reflective experiences for the sub-group of HDR students in this sample supported the scores and profiles measured by the BRUMS.

The challenge of feeling competent in an academic environment may be explained by the big-fish-little-pond effect (BFLPE) [56, 57]. Emphasising social comparison theory [58], and accentuating the multidimensional nature of self-concept [59], the BFLPE proposes that academic self-concept is shaped by evaluative comparisons with peers who provide a favourable or unfavourable frame of reference by which to gauge level of academic competence. While commonly investigated in school-aged children and adolescents, the BFLPE has been found to extend to higher education settings cross-culturally [60]. Social isolation may hinder the development of a positive academic self-concept due to segregation from relative target models. HDR students already boast high-level academic skills and abilities although may inadvertently rank themselves against practised supervisors to gain verification. Such misdirected efforts would only serve to reinforce an unfavourable evaluation of academic competency, and further strengthen the perception of being *a little fish in a big pond*. This notion is supported by a meta-analysis by Gerber et al., which found that individuals were 76% more likely to make upward comparisons in lieu of access to equivalent peers [61]. Of note, academic self-confidence is reciprocally related to academic accomplishment [62], and has implications for student well-being, making salient the importance of providing adequate supports [63].

### Strengths and limitations

The adequate sample of HDR students and mixed-methods approach implemented can be considered strengths of our study. A priori power analysis was calculated using G*Power [64]. Results indicated that the sample size of 231 was in excess of that required to achieve 80% power at a significance level of *p*< .05. However, we would wish to acknowledge some potential limitations. The research was carried out at one regional university in Australia. While this provides an important snapshot of mental health and wellbeing of HDR students, the findings need to be replicated to understand if this picture is generalizable beyond this context.

However, what the findings do indicate is the need for further attention to be paid to support the mental health and wellbeing needs of HDR students. HDR students need to be more involved in a flourishing research community to support social connection and opportunities post-PhD. Given the importance of supervisor relationships, continued attention needs to be paid to fostering positive and supportive supervisory teams, with supports and training available for supervisors to facilitate this. As such, support for positive mental health and wellbeing needs to be an increased focus for universities and clear steps including HDR students being more involved in a flourishing research community need to be more clearly taken in order to support social connection and feelings of competence amongst the current HDR student community.

## Supporting information

S1 Data (SAV)
